# Knowledge, Attitude and Implementation of Evidence-Based Practice among Physiotherapists Working in the Kingdom of Saudi Arabia: A Cross-Sectional Survey

**DOI:** 10.3390/healthcare8030354

**Published:** 2020-09-22

**Authors:** Fatmah Hasani, Joy C. MacDermid, Ada Tang, Michelle Kho, Ahmad H. Alghadir, Shahnawaz Anwer

**Affiliations:** 1 School of Rehabilitation Science, McMaster University, Hamilton, ON L8S 1C7, Canada; fatmahhasani@gmail.com (F.H.); macderj@mcmaster.ca (J.C.M.); atang@mcmaster.ca (A.T.); khome@mcmaster.ca (M.K.); 2 Clinical Research Lab, Hand and Upper Limb Centre, St. Joseph’s Health Centre, Toronto, ON M6R 1B5, Canada; 3 Rehabilitation Research Chair, College of Applied Medical Sciences, King Saud University, Riyadh 11451, Saudi Arabia; aalghadir@hotmail.com; 4 Department of Building and Real Estate, Hong Kong Polytechnic University, Kowloon, Hong Kong

**Keywords:** barriers, physical therapy, survey, rehabilitation, patient care, decision-making, healthcare

## Abstract

The current study aimed to investigate knowledge, attitudes, and implementation of evidence-based practice among physiotherapists working in Saudi Arabia. A sample of physiotherapists working in various outpatient settings in Saudi Arabia participated in this survey. Sixty-four therapists (30 females, 34 males) completed a 28-item survey questionnaire. Approximately half of the participants indicated that evidence-based practice was useful and important for clinical practice. About 60% of the participants reported that they have adopted the evidence-based practice on a regular basis. Participants who had a membership in a physiotherapy organization and had advanced degrees showed more positive attitudes (t = −2.31, *p* = 0.02 and t = −2.15, *p* = 0.04, respectively) and greater levels of knowledge (t = −2.32, *p* = 0.02 and t = −3.86, *p* = 0.001, respectively) regarding evidence-based practice terminology. Furthermore, place of training (trained overseas) was associated with a positive attitude towards literature findings (t = 2.26, *p* = 0.03). The results of this study demonstrated that participants reported that evidence-based practice had not been extensively implemented, despite positive attitudes regarding its implementation among physiotherapists practicing in Saudi Arabia.

## 1. Introduction

The idea of evidence-based practice (EBP) has gained importance in physiotherapy clinical practice over the last two decades [1,2,3,4,5]. EBP has become the gold standard in clinical practice across various fields, including medical, rehabilitation and allied health science [6,7,8]. Sackett et al. [9] have defined EBP as a rational, specific, and thoughtful use of the best available current evidence in making clinical decisions regarding individual patient care. According to the Sicily statement on EBP, clinical decisions, should be made based on current, valid, relevant, and best evidence [10].

Knowledge of how various healthcare professionals implement EBP can determine the gaps in contemporary clinical practice and advance professional growth at an individual or group level, at a larger professional body. A recent systematic review indicated that most physiotherapists showed a positive point of view about EBP; however, they emphasize the need for improved knowledge, skills, and behavior regarding EBP [4]. In addition, physiotherapists have identified various barriers to using EBP, including lack of time, lack of support from their employer, inability to understand statistics, lack of resources, lack of generalizability of results, and lack of interest [4]. More recently, Alshehri et al. [11] identified a significant gap in the knowledge and application of the concept of EBP among physiotherapists in Saudi Arabia. Additionally, most of the physiotherapists indicated inadequate formal training in EBP as the major problem for implementing EBP in clinical practice [11]. Similarly, Alqahtani et al. [12] reported that approximately 60% of the physiotherapists participants from Saudi Arabia were not aware of how to implement and use EBP in their everyday practice. A previous study highlights the urgent need for the required changes in the current educational system to improve EBP implementation in Saudi Arabia [7]. Similarly, other studies recommended improving knowledge and skills to more successfully implement EBP use among healthcare professionals in Saudi Arabia [13,14]. 

Physiotherapy education in Saudi Arabia was started relatively late compared to many countries [15,16,17,18], as the first bachelor’s program in physiotherapy was started in the year 1985 at King Saud University [19]. In the last two decades, significant advancement has been seen at several universities and physiotherapy graduate programs in Saudi Arabia resulting in the increase of physiotherapy programs from 6 to 16 [15]. Previous studies have suggested that the expansion of EBP implementation is needed in developing countries in order to achieve high quality patient care [20,21,22]. Although the idea of EBP is somewhat new in Saudi Arabia, strategies are in place to change practice from the dependence on professional opinions to the utilization of research-based evidence [23]. These include the advancement of EBP skills, such as appraising, interpreting, accessing, and using scientific literature, in all entry-level medical education programs [24,25,26,27]. However, these changes were mostly restricted to medical education programs [24,25,27]. Therefore, the present study aimed to investigate knowledge, attitudes, and implementation of EBP among physiotherapists working in Saudi Arabia. 

## 2. Materials and Methods 

### 2.1. Survey Development

A self-administered cross-sectional questionnaire-based survey was used for the current study, which was designed by the authors (J.C.M. and F.H.) and comprised of a 28-item questionnaire to assess knowledge, attitudes, and implementation of EBP [28,29,30]. Recently, Shi et al. [30] reported a high internal consistency (Cronbach’s alpha = 0.85) and supported the construct validity of a 4-factor structure of modified evidence-based practice- knowledge, attitudes, behavior and decisions/outcomes questionnaire among physiotherapists and occupational therapist. Pilot testing was conducted in the current study to assess the content validity and to examine and acknowledge any probable issues regarding clarity, readability, and grammatical structure of each item. A purposive sample of 9 experienced physiotherapists from Canada (*n* = 2) and Saudi Arabia (*n* = 7) participated in the pilot testing. Individuals involved in the pilot testing were therapists representing a broad spectrum of practice areas, including pediatrics (*n* = 1), orthopedics (*n* = 1), in-patient care (*n* = 1), out-patient clinic (*n* = 2), academics (*n* = 3), and hand therapy (*n* = 1). Internal consistency scores of the pilot survey yielded values of Cronbach’s alpha = 0.5 (attitude), 0.7 (confidence and perceived skills), 0.7 (knowledge), and 1 (hand outcome measures) [31]. Additionally, the internal consistency reliability scores were assessed for the current survey questionnaire response of 64 participants. Internal consistency scores of total participants yielded values of Cronbach’s alpha = 0.80 (attitude), 0.87 (confidence and perceived skills), 0.87 (knowledge), and 0.95 (hand outcome measures) [31]. A summary of survey content is presented in Table 1. 

The survey questionnaire had three sections and was designed to be completed in 15–20 min. The first section assessed physiotherapists’ responses about EBP, the second section examined caseload, and the third section contained demographic questions. Data included in the questionnaire were based on the following items: (1) Attitudes and beliefs about EBP, designed to explore the extent to which physiotherapists viewed the usefulness of certain statements in their practice, including the application of research findings in practice and the application or implementation of EBP in practice. (2) Perceived skills and confidence in EBP, which assessed physiotherapists’ confidence to apply EBP and engage in clinical decision-making, and (3) integration of EBP, designed to assess physiotherapists’ opinions toward the integration of EBP in academic training. (4) Adoption of EBP in daily clinical practice, and (5) knowledge about EBP terminology (e.g., systematic review, relative risk, publication bias, and confidence interval) [31,32].

### 2.2. Participants

A pool of physiotherapists who were potential respondents to the survey were invited. However, because the availability of physiotherapy educational programs in Saudi Arabia is relatively new, we expected that most participants would be younger in age (i.e., 30-years-old or younger) [11]. The study was approved by the Hamilton Integrated Research Ethics Board (project number 13-647-S). All participants provided written informed consent before completing the survey.

### 2.3. Survey Administration 

Adequate numbers of the survey questionnaire were given to senior therapists at five public hospitals in the major cities of Saudi Arabia to distribute to staff physiotherapists. To minimize selection bias, these senior hospital therapists generated lists of random names from their pool of staff physiotherapists and sent them paper surveys. Each survey included a cover letter with study objectives and description. A follow-up reminder was made personally with all senior therapists after one week, regardless of whether the questionnaires were completed. The completed surveys were returned in an envelope to senior hospital therapists, who subsequently delivered them to the first author. 

### 2.4. Statistical Analyses

Initially, the items in the survey questionnaire were evaluated using a 5-point Likert scale (e.g., attitude response set), which included the categories “very useful”, “somewhat useful”, “no opinion”, “not very useful”, and “not useful at all”. Additionally, items using a 6-point Likert scale (e.g., confidence response sets), included the categories “high”, “a lot”, “moderate”, “somewhat”, “a little”, and “not at all”. Since too many available categories of a Likert scale may obscure rather than define the purpose of respondent, reducing the number of response categories by collapsing across responses may improve the outcomes of analysis [32]. Therefore, the last three categories of attitude response sets were combined to the category “not useful.” Similarly, the confidence response sets were collapsed to the final three categories, “high” “moderate,” and “not much.” The “high” and “a lot,” categories were combined as “high” and “somewhat “, “a little”, and “not at all” were combined as “not much”. Each response was assigned a numerical value, so that parametric statistics may be used for Likert data [33]. A high score indicates more positive response. Descriptive statistics, including the frequency and percentage, were calculated to characterize the study participants. An independent *t*-test with 95% confidence interval was used to compare the groups (i.e., age (<30 vs. >30 years), education level (undergraduate vs. graduate), place of clinical training (Saudi Arabia vs. abroad), and professional membership (yes vs. no) and physiotherapists’ responses regarding EBP). The current study hypothesizes that an older, graduate, abroad degree holder, and who is a member of any professional body would give more positive responses regarding knowledge and the use of EBP. The data were transferred to Excel spreadsheets and then imported to IBM SPSS version 22 for statistical analysis. The results were considered statistically significant if *p* < 0.05.

## 3. Results

### 3.1. Participants’ Characteristics

A total of 64 physiotherapists from different regions in Saudi Arabia who were national and international practitioners in Saudi Arabian hospitals completed the survey. Participants’ characteristics are described in Table 2. Thirty (47%) participants were female, and the majority were from Jeddah City (*n* = 52, 81%). There were also more Saudi participants (*n* = 47, 73%) than international participants (*n* = 17, 26%). Most participants were in the youngest age group (20–29 years, *n* = 43, 67%) and held a bachelor’s degree (*n* = 49, 77%). In addition, 63% (*n* = 40) of the participants practiced in government hospitals, and more than half (*n* = 36) were members of physiotherapy organizations. The majority (*n* = 33, 52%) of the participants had worked between one to five years.

### 3.2. Attitudes and Opinions

Physiotherapists who work in Saudi Arabia showed positive attitudes and opinions about EBP. Specifically, 45% (*n* = 29) viewed EBP as very useful, and 47% (*n* = 30) viewed EBP as useful to some extent, whereas only 8% (*n* = 5) reported EBP as not very useful. In addition, 55% (*n* = 35) of participants reported that evidence-based literature findings were somewhat useful, while 31% *(n* = 20) and 14% (*n* = 9) of participants reported very useful and not very useful, respectively. A difference was noted between participants’ attitudes toward literature findings and their degrees, place of training, and professional memberships (t = −2.15, *p* = 0.04; t = −2.26, *p* = 0.03; and t = −2.31, *p* = 0.02, respectively) (Table 3).

### 3.3. Confidence and Perceived Skills

While 59% (*n* = 38) of participants reported a high confidence in EBP uses, 33% (*n* = 21) and 8% (*n* = 5) of participants reported moderate and not much confidence, respectively. Moreover, while 70% (*n* = 45) of participants reported a high confidence in decision making, 30% (*n* = 19) of participants reported moderate confidence, respectively. Participants who earned advanced academic degrees (i.e., higher than a bachelor’s degree) reported higher confidence in their EBP skills (t = −2.36, *p* = 0.02) (Table 4). 

### 3.4. Perceived Knowledge

Proportions of self-reported knowledge of EBP terms (confidence interval, publication bias, relative risk, and systematic review) is given in Table 5. Fewer than half (*n* = 29, 45%) of participants reported that they completely understood both the terms “relative risk” and “systematic review,” and about 42% of the participants partially understood these terms. However, fewer participants understood the terms “confidence interval” (*n* = 10, 16%) and “publication bias” (*n* = 11, 17%). A significant difference was found between participants’ knowledge regarding EBP terms and their degrees or professional memberships (t = −3.86, *p* = 0.001 and t = −2.32, *p* = 0.02, respectively) (Table 6). There were no differences between national and international therapists in terms of their level of understanding of search terminologies.

### 3.5. EBP Implementation

Only a few participants reported implementing evidence-based research in their practice, as 11% (*n* = 7) reported doing so daily, and 16% (*n* = 10) reported doing so regularly. The frequency with which participants read literature findings or looked for scientific evidence ranged from 3% (*n* = 2) who did so daily, 34% (*n* = 22) who did so weekly, and 33% (*n* = 21) who did so monthly. Most participants reported attending few courses or workshops per year (≤3) (*n* = 39, 61%), while 33% (*n* = 21) attended four to six courses annually. Approximately 48% of participants had access to the Internet in their workplace, but only 38% (*n* = 24) used it to access scientific evidence. In contrast, 39% (*n* = 25) of participants reported that they did not have access to the internet in their workplace, but they did search for evidence at home.

## 4. Discussion

The results of the current study provide an outline of the present self-reported attitudes toward and utilization of EBP among physiotherapists practicing in Saudi Arabia. Overall, results indicated that physiotherapists exhibit a positive attitude toward EBP usage. Further, the positive attitudes of those with higher degrees and membership of physiotherapy organizations were more toward literature findings than EBP. The results of the current study share some similarities and differences with other studies conducted among physiotherapists and other healthcare professionals in Saudi Arabia. For instance, a recent study has investigated the knowledge, attitude and behaviors toward EBP of physiotherapists working in Saudi Arabia [11]. They concluded a significant gap in knowledge about concept and process of implementing EBP dimensions, including knowledge, attitude and outcome/decisions [11]. Another study reported poor level of knowledge and attitudes towards EBP implementation and uses among healthcare physicians in Saudi Arabia [7]. A cross-sectional study of 288 physicians from Saudi Arabia revealed that they had positive attitudes toward EBP, however, it was not associated with their knowledge and awareness [34]. Many countries such as Australia [3], Spain [35], UK [36], and the USA [5] have already included EBP topics in their undergraduate or graduate physiotherapy curricula. In contrast, many developing countries such as Saudi Arabia are yet to include the important topic of EBP in their undergraduate or graduate physiotherapy curricula.

### 4.1. Attitudes toward EBP

Our findings indicated that practicing physiotherapists in Saudi Arabia have positive attitudes toward EBP and show a keen interest in reading literature findings for collecting evidence. A previous study reported high levels of positive attitude toward EBP among various professionals, including physicians, nurses, physiotherapists, occupational therapists and psychologists [37]. Another study reported a positive attitude toward EBP across health professionals [38]. Similarly, other studies also reported higher positive attitudes toward EBP in certain healthcare workers such as nurses [39,40], occupational therapists [41], physicians [28,42,43,44], and physiotherapists [3,45]. Demographic data in the current study showed that attitudes toward EBP did not differ for younger or older physiotherapists. This finding contrasts with a previous study in which Jette et al. [5] reported that young American physiotherapists were more likely to report positive attitudes toward EBP. This lack of difference between EBP attitudes and age in the present study could be a result of small sample size or career length, given that physiotherapy bachelor’s programs are relatively new in most geographical regions of Saudi Arabia (i.e., the expansion of bachelor’s programs started in 2003), and most participants were relatively young (i.e., in their 20s).

On the other hand, our results suggest that physiotherapists with post-graduate academic degrees tend to have more positive attitudes regarding the importance of scientific literature, and they view it as helpful in their practice. They are also more likely to have confidence in EBP skills than those with lesser academic qualifications. Previous studies have identified that postgraduate education is vital for physiotherapists to improve their attitude, knowledge, and skills of using EBP in daily clinical practice [46,47,48]. Other studies reported a positive relationship of higher academic degrees with knowledge of EBP [49], better implementation of EBP [50], and more positive attitudes towards research utilization [51,52]. Similarly, Alshehri et al. [11] reported a significant association between educational level and positive attitude towards EBP among physiotherapists in Saudi Arabia. We believe that the impact of higher education on positive attitude and opinion about EBP reflects the degree of emphasis on the reading of scientific literature and research skills in programs offering advanced degrees compared to those that only provide an undergraduate-level training. Similarly, previous studies have also found evidence of positive attitudes regarding the importance of scientific literature among physiotherapists [5,53], physicians [54], and nurses [55]. Therefore, the present study suggests that EBP education and training during postgraduate education might have the greatest influence on improving EBP attitudes and skills among postgraduate professionals than undergraduate professionals. 

Physiotherapists who trained outside the country reported more positive attitudes towards the importance of literature findings. One potential reason for this finding is that there is a lack of research in the disability field in Saudi Arabia, which may decrease the likelihood of being able to see the value of keeping up to date with literature findings [56]. While many universities in India [57] and the United Arab Emirates [58] include subjects related to research methods and EBP in undergraduate physiotherapy curricula, these subjects are not included at undergraduate level in most of the Saudi universities [15]. This was reflected in the attitudes and opinions of foreign trained physiotherapists towards EBP and literature findings. However, the relationship between place of training and more positive attitudes towards the importance of literature finding was not studied previously. 

In the current study, therapists who had a membership in physiotherapy professional organizations reported more positive attitudes regarding the importance of literature findings, and they viewed it as helpful compared to those who did not belong to physiotherapists associations. For example, physiotherapy professional organizations provide an important platform for the continuing professional education program on EBP and they give access to many peer reviewed journals. Therefore, membership was an important factor to improve attitude, knowledge, and skills about EBP among physiotherapists [52,59]. Additionally, professional organizations may provide many learning opportunities such as online or face-to-face training courses about the fundamentals of EBP, which is very much important for improving EBP skills [8]. This finding supports the notion that involvement in professional associations and continuing education opportunities is essential in improving skills, knowledge, and behaviors [5]. Involvement in professional organizations may be associated with more dissemination of information about EBP, attendance at professional meetings that promote EBP, or access to journals that positively affect knowledge and attitudes regarding EBP. Indeed, therapists who belonged to physiotherapy organizations demonstrated greater knowledge of most EBP terms. However, the relationship between professional organization membership and the absence or presence of curricular EBP for positive attitudes towards EBP implementation by physiotherapists remains unknown.

### 4.2. Perceived Knowledge, Confidence, and Skills

In terms of perceived knowledge regarding EBP terminology, previous work has found that the technical terms used across the literature are often challenging to understand [28]. In the present study, participants with post-graduate-level education reported a complete ability to understand EBP terms, such as “confidence interval” and “publication bias,” as compared to undergraduate level education, who reported that they were only able to understand such terms somewhat or not at all. This finding is consistent with previous research demonstrating that individuals with graduate training are expected to be more open to research and technical terms used in the field [5,53]. Since postgraduate students have extensive knowledge regarding research methods, they have shown improved confidence in the use of research skills during clinical practice. Similarly, other studies reported an association between postgraduate education and greater EBP knowledge and skills [60,61,62,63]. Moreover, another study reported a positive correlation between highest qualification and the greater use of literature in decision making by Malaysian physiotherapists [64]. A previous study also reported that physiotherapists with postgraduate qualification are expected to have a greater extent of research and critical appraisal skills than undergraduate physiotherapists [65]. A systematic review reported a positive association between a higher academic level and more positive attitudes toward the use and adoption of EBP [11]. Therefore, participants who do not have a postgraduate degree are less likely to use EBP in their clinical practice owing to a lack of knowledge and skills about EBP [64]. 

EBP supports the use of clinical decision-making to improve the quality of patient care [10]. Researchers believe that clinical decision-making is influenced by the knowledge and experience [66], such that greater experience has been shown to facilitate quicker decisions [67] and foster pattern recognition of information [68,69]. Previous studies have reported that most physiotherapists base their clinical decisions on the knowledge they acquired during their entry-level training [3,5,70]. In the current study, participants varied as to whether they had completed training sessions about EBP in their foundation programs. Specifically, only 9% of the respondent reported having engaged completely in such sessions during training, whereas the majority (34%) reported having had only moderate training engagement in EBP. Moreover, participants with graduate level degrees reported having received training in EBP more often than those with undergraduate degrees. These findings are consistent with those of a previous study, which also found that Physiotherapists in Saudi Arabia reported a lack of formal training in EBP [27,37]. In contrast, other studies, for example, Jette et al. [5] reported that 67% of the participants received formal training about research literature, and Ramırez-Velez et al. [71] reported that 88% of the respondents had obtained basic knowledge of EBP. Although clinical decision-making improves with practice, time alone does not ensure experiential learning [72]. The combination of experience and skills, such as critical thinking and patient communication, is required to make effective clinical decisions [73]. Previous research has thus recommended that EBP related concepts be included in both undergraduate and graduate curricula [11]. Therefore, we recommend including an introductory session and comprehensive lectures about EBP in undergraduate and postgraduate physiotherapy curriculum in Saudi Arabia.

### 4.3. EBP Implementation

The current study had a promising finding in that most participants reported having accessed evidence-based research on a weekly or monthly basis, and 48% had access to the Internet at home or at work. In addition, an unexpected association was found between Internet access at home or work and physiotherapists’ nationality. Specifically, international physiotherapists accessed the Internet more often at work (44%) than did domestic physiotherapists (35%) to search for evidence-based research. Research has demonstrated the challenging nature of EBP implementation in clinical practice settings [74,75]. Although most participants (89%) had positive attitudes toward EBP and believed it was necessary, only a few reported having implemented EBP in their daily practice (11%) or on a regular basis (16%). This finding is consistent with that of other studies, which have indicated that positive attitudes about EBP do not necessarily translate into implementation [29]. A previous systematic review indicates that positive attitudes about EBP were not correlated with the persistent and adequate use of EBP by physiotherapists [4]. Similarly, a previous study reported a trend of lower implementation scores than other dimensions of EBP among physicians, nurses, physiotherapists, occupational therapists and psychologists [37]. In this vein, Condon et al. [76] have recommended that it is crucial to identify the information needs of physiotherapists prior to implementation of EBP.

### 4.4. Implications

Overall, this study was mainly descriptive, and it highlighted the attitudes and behaviors of physiotherapists toward EBP and areas of strength and weakness in the current knowledge and skills of physiotherapists in Saudi Arabia. This study can serve as a foundation for further EBP studies among physiotherapists in Saudi Arabia. Thus, our findings have clinical, academic, and research implications, which are likely to interact. In the current study, only 10% of the respondents reported having completely or somewhat learned about EBP in their foundation training. This finding is crucial in that it calls for the education community to update the curriculum of the physiotherapy program and to standardize the physiotherapy education to include topics such as EBP, and critical appraisal, which may transfer the EBP from belief to adoption in physiotherapy practice in Saudi Arabia. Since the present study identified that limited time and poor skills were the major perceived barriers to the implementation of EBP, continuing education at clinical sites or local practice should focus on improving practitioners’ skills and efficiency in searching for resources. Additionally, clinical administrators should be encouraged to increase the availability of computer access to research databases and to provide time for practitioners to retrieve literature. Practitioners could increase the amount of communication about the research findings among their colleagues (e.g., assigning sessions for seminars or case studies). Moreover, there were obstacles in work environments such as a lack of motivation to change, and communication that should be discussed in depth and action should be taken to improve productivity in the workplace. Consequently, it is recommended that quality improvement projects be developed to discuss and facilitate the identified issues.

### 4.5. Limitations

The current study has some limitations. First, this study relied on self-reported data using the questionnaire, and many concepts were subject to personal bias. Additionally, such questionnaires might be subject to untruthful answers, recall bias, misinterpretation of questions by the respondents and missing data [77]. Second, the study was characterized by a small sample size. Third, we could not assess for response bias because the data for non-responders were not available; therefore, the generalizability outside those employed in hospital settings is limited. Fourth, the similarity and differences between physiotherapists working in governmental settings and those in private settings was unclear. While the current study does provide insight into the perceptions of EBP among physiotherapists across a broad sample, i.e., new/experienced clinicians, members/non-members of physiotherapists professional associations, national/international clinicians, a comprehensive study involving a wider range of participants from different cities, different age groups and other practical settings including private and specialized clinics could reveal data that are more conclusive. Due to presence of many confounding variables such as work experience (e.g., novice to expert practitioners), highest qualifications (e.g., postgraduate masters or doctorate), more studies are required on a larger sample for further validation of these results. 

## 5. Conclusions

The present study indicated that self-reported EBP has not been widely implemented, despite the positive attitudes towards its use among physiotherapists practicing in Saudi Arabia. This study highlights the difference between EBP knowledge between those trained at an undergraduate level and those at a graduate level. This is an important finding and has implications to influence the inclusion of EBP concepts in undergraduate curricula of physiotherapy in Saudi Arabia. These findings, although important, do not fully capture the full spectrum of physiotherapists’ attitudes and skills towards using EBP in Saudi Arabia. These findings may help redesign the curriculum, post-professional training or interventions that might be needed to encourage or facilitate the uptake of EBP in Saudi Arabia.

## Figures and Tables

**Table 1:** Survey content.

Variables	Total Number of Items
Attitudes or opinions about integration of evidence-based practice in academic training	2
Perceived confidence in evidence-based practice skills and decision-making	2
Integration of evidence-based practice as learning tools	1
Perceived skills of performing physical assessment	1
Adoption of evidence-based practice	4
Knowledge about evidence-based practice terminology (confidence interval, publication bias, relative risk, systematic review)	1
Perceived barriers and work limitation	2
Assessment regarding participants’ caseload	4
Respondents’ characteristics	11
Total	28 questions

**Table 2:** Participant characteristics.

Characteristics	*n*	%
Gender		
Male	34	53
Female	30	47
Age group		
20–29 years	43	67
30–39 years	17	27
40–49 years	2	3
≥ 50 years	2	3
Geographical area of practice		
Jeddah	52	81
Makkah	1	2
Riyadh	3	5
Other	8	12
Nationality		
Saudi	47	73
Non-Saudi	17	27
Current practice position		
Senior	12	19
Junior	30	47
Intern	11	17
Other	11	17
Highest professional degree		
Diploma	7	10
Bachelor	49	77
Master	7	12
Doctoral	1	1
Post-doctoral	0	0
Place of training		
Saudi Arabia	47	73
Abroad	17	27
Type of practical setting		
Government hospital	40	62
Private hospital	3	5
University educational hospital	20	30
Other	2	3
Years licensed		
Less than a year	12	19
1–5 years	33	51
6–10 years	12	19
11 years and more	7	11
Clinical certified specialty		
No	48	75
Yes	16	25
Membership in professional organization (Saudi Physical Therapists Association)		
Yes	36	56
No	28	44

**Table 3:** Comparison of demographic characteristics and attitudes toward evidence-based practice (EBP) and literature findings.

Independent Variables	Category	EBP Attitude					Literature Findings				
		Mean (SD)	t	*p*	Mean Difference	†95% CI	Mean (SD)	t	*p*	Mean Difference	†95% CI
Age	<30	2.35 (0.61)	−0.47	0.64	−0.08	−0.42 to 0.26	2.14 (0.60)	0.56	0.58	0.10	0.05 to 0.25
	>30	2.43 (0.68)					2.24 (0.77)				
Highest degree	Undergraduate	2.32 (0.64)	−1.83	0.07	−0.43	−0.90 to 0.04	2.11 (0.62)	2.15	0.04 *	0.52	0.24 to 0.74
	Graduate	2.75 (0.46)					2.63 (0.74)				
Place of training	Saudi	2.30 (0.66)	−1.65	0.10	−0.29	−0.64 to 0.06	2.06 (0.60)	2.26	0.03 *	0.41	0.27 to 0.65
	Abroad	2.59 (0.51)					2.47 (0.72)				
Membership	No	2.36 (0.73)	−0.20	0.84	−0.03	−0.29 to 0.35	1.96 (0.74)	2.31	0.02 *	0.37	0.25 to 0.69
	Yes	2.39 (0.55)					2.33 (0.54)				

†95% Confidence interval of the difference; * Statistically significant if *p* < 0.05.

**Table 4:** Comparison of demographic characteristics and confidence with evidence-based practice (EBP) uses and clinical decision-making skills.

Independent Variables	Category	EBP Uses					Decision-Making				
		Mean (SD)	t	*p*	Mean Difference	†95% CI	Mean (SD)	t	*p*	Mean Difference	†95% CI
Age	<30	2.42 (0.66)	−0.47	0.64	−0.08	−0.42 to 0.26	2.63 (0.49)	−0.56	0.58	−0.10	−0.45 to 0.25
	>30	2.71 (0.56)					2.86 (0.36)				
Highest degree	Undergraduate	2.45 (0.66)	−2.36	0.02 *	−0.55	−1.02 to −0.09	2.68 (0.47)	−1.13	0.26	−0.10	−0.54 to 0.15
	Graduate	3.00 (0.00)					2.88 (0.35)				
Place of training	Saudi	2.45 (0.69)	−1.44	0.16	−0.26	−0.62 to 0.10	2.66 (0.48)	−1.26	0.21	−0.16	−0.42 to 0.10
	Abroad	2.71 (0.47)					2.82 (0.39)				
Membership	No	2.39 (0.69)	−1.36	0.18	−0.22	−0.10 to 0.54	2.68 (0.48)	−0.37	0.71	−0.04	−0.19 to 0.27
	Yes	2.61 (0.60)					2.72 (0.45)				

†95% Confidence interval of the difference; * Statistically significant if *p* < 0.05.

**Table 5:** Proportions of therapists’ self-reported knowledge of evidence-based practice (EBP) terms (confidence intervals, publication bias, relative risk, and systematic review).

Independent Variables	Category	Confidence Interval			Publication Bias			Relative Risk			Systematic Review		
		Somewhat *n* (%)	NO *n* (%)	YES *n* (%)	Somewhat *n* (%)	NO *n* (%)	YES *n* (%)	Somewhat *n* (%)	NO *n* (%)	YES *n* (%)	Somewhat *n* (%)	NO *n* (%)	YES *n* (%)
Age	<30	25 (58)	13 (30)	5 (12)	17 (40)	19 (44)	7 (16)	19 (45)	4 (10)	19 (45)	18(43)	6 (14)	18 (43)
	≥30	10 (48)	6 (29)	5 (23)	10 (48)	7 (33)	4 (19)	8 (38)	3 (14)	10 (48)	8 (38)	2 (10)	11 (52)
Highest degree	Undergraduate	33 (59)	18 (32)	5 (9)	26 (46)	25 (45)	5 (9)	25 (45)	7 (13)	23 (42)	25 (45)	8 (15)	22 (40)
	Graduate	2 (25)	1 (12)	5 (63)	1 (12)	1 (12)	6 (75)	2 (25)	0 (0)	6 (75)	1 (13)	0 (0)	7 (87)
Place of training	Saudi	27 (57)	14 (30)	6 (13)	21 (45)	21 (45)	5 (10)	19 (41)	6 (13)	21 (46)	18 (39)	7 (15)	21 (46)
	Abroad	8 (47)	5 (29)	4 (24)	6 (35)	5 (30)	6 (35)	8 (47)	1 (6)	8 (47)	8 (47)	1 (6)	8 (47)
Membership	Yes	18 (50)	9 (25)	9 (25)	14 (39)	15 (42)	7 (19)	15 (42)	1 (3)	20 (55)	13 (36)	2 (6)	21 (58)
	No	17 (61)	10 (36)	1(3)	13 (46)	11 (39)	4 (14)	12 (45)	6 (22)	9 (33)	13 (48)	6 (22)	8 (30)

NO = did not understand; YES = completely understand.

**Table 6:** Comparison of demographic characteristics and knowledge of evidence-based practice (EBP) terms (confidence intervals, publication bias, relative risk, and systematic review).

Independent Variables	Category	Knowledge of EBP terms				
		Mean (SD)	t	*p*	Mean Difference	†95% CI
Age	<30	2.05 (0.48)	−0.66	0.51	−0.09	−0.38 to 0.19
	>30	2.14 (0.62)				
Highest degree	Undergraduate	1.99 (0.48)	−3.86	0.001 *	−0.69	−1.06 to −0.34
	Graduate	2.69 (0.46)				
Place of training	Saudi	2.03 (0.50)	−1.15	0.25	−0.17	−0.47 to 0.13
	Abroad	2.21 (0.60)				
Membership	No	1.90 (0.51)	−2.32	0.02 *	−0.31	−0.04 to 0.57
	Yes	2.21 (0.51)				

†95% Confidence interval of the difference; * Statistically significant if *p* < 0.05.
